# Predicting non-response to multimodal day clinic treatment in severely impaired depressed patients: a machine learning approach

**DOI:** 10.1038/s41598-022-09226-5

**Published:** 2022-03-31

**Authors:** Johannes Simon Vetter, Katharina Schultebraucks, Isaac Galatzer-Levy, Heinz Boeker, Annette Brühl, Erich Seifritz, Birgit Kleim

**Affiliations:** 1 Department of Psychiatry, Psychotherapy and Psychosomatics, Psychiatric University Hospital, University of Zurich, Lenggstrasse 31, 8032 Zurich, Switzerland; 2 Vagelos School of Physicians and Surgeons, Department of Emergency Medicine, Columbia University Medical Centre, New York, NY USA; 3 Department of Psychiatry, Columbia University, New York, NY USA; 4 Department of Psychiatry, NYU Grossman School of Medicine, New York, NY USA

**Keywords:** Psychology, Risk factors

## Abstract

A considerable number of depressed patients do not respond to treatment. Accurate prediction of non-response to routine clinical care may help in treatment planning and improve results. A longitudinal sample of N = 239 depressed patients was assessed at admission to multi-modal day clinic treatment, after six weeks, and at discharge. First, patient’s treatment response was modelled by identifying longitudinal trajectories using the Hamilton Depression Rating Scale (HDRS-17). Then, individual items of the HDRS-17 at admission as well as individual patient characteristics were entered as predictors of response/non-response trajectories into the binary classification model (eXtremeGradient Boosting; XGBoost). The model was evaluated on a hold-out set and explained in human-interpretable form by SHapley Additive explanation (SHAP) values. The prediction model yielded a multi-class AUC = 0.80 in the hold-out set. The predictive power for the binary classification yielded an AUC = 0.83 (sensitivity = .80, specificity = .77). Most relevant predictors for non-response were insomnia symptoms, younger age, anxiety symptoms, depressed mood, being unemployed, suicidal ideation and somatic symptoms of depressive disorder. Non-responders to routine treatment for depression can be identified and screened for potential next-generation treatments. Such predictors may help personalize treatment and improve treatment response.

## Introduction

Depression is a severely debilitating disorder with significant personal costs and economic impact^1–4^. Effective treatments for depression have been developed and successfully disseminated, including treatments that specifically target severe and chronic depression^5–7^. Despite the effectiveness of these treatments on average, they are not equally effective for all depressed patients. In fact, there is evidence that some depressed patients may not respond to standard treatments, i.e. comprising a subgroup of treatment-resistant patients warranting further clinical characterization and attention^8–10^. Identifying those patients who most likely benefit from a given treatment is paramount and key to developing an efficient and personalized depression treatment^1,11^. This may also help in treatment planning and improving results^12^ as well as for resource allocation. Further, with new classes of therapies for treatment resistant depressive patients, early targeted treatment can reduce burdensome and costly trial-and-error approaches.

There is evidence that disaggregating the heterogeneity in the depression diagnosis can aid in understanding the likely treatment course^13,14^. Based on these observations, data-driven techniques have been used to cluster patients with regard to their symptom patterns in association with their course trajectories^15,16^ resulting in promising findings including, for instance, predicting worse outcomes in anxiety disorder by anhedonia^16^, dysphoria, suicidality, anxiety, and early onset^17^, sad mood and concentration difficulties^18^, information on comorbidities^19,20^, and initial treatment response to antidepressants^21^. The mentioned studies were based on clinical trial data^18,21^, small sample sizes^16^, or retrospective information^17,19^. Real world samples are needed to build models that are relevant to clinic based samples (e.g.,^20,22,23^).

When applied to clinical features and other data available at baseline to develop prediction models of subsequent depression course and treatment response, machine learning (ML) is promising^24–26^. These methods identify patterns of information to predict outcomes at the individual patient level^27^ and can detect complex high-dimensional interactions, for instance in major depressive disorder (MDD)^28–30^.

Here, we focus on a group of depressed patients commonly presenting to psychiatric care, attaining multimodal depression treatment in a day clinic of a large urban psychiatric hospital in Switzerland. Patients were severely ill, with significant impairment in psychosocial functioning, mostly long pre-treatment absences at workplaces, high levels of suicidal ideation and drop-out rates^31^. Day clinic treatments for acute care are comparable to inpatient programs in terms of the intensity of the delivered multimodal treatment programs, with the difference that patients return home in the evenings and over the weekends^32^ and have become increasingly relevant in the context of reducing costs of inpatient care^33^ and particular advantages of the setting, e.g., intensive transfer into real life.

This longitudinal observational study in the context of a multimodal treatment aimed to (i) identify latent subgroups of treatment response trajectories amongst depressed day clinic patients, using Latent Growth Mixture Models (LGMM)^34^ with standard clinical data that is readily available in most clinic settings. Given that such subgroups can be identified, our second aim was to (ii) detect early predictors for treatment outcome. Identification of such predictors could help optimize treatment planning and improve treatment outcomes.

## Methods

### Participants

We analysed data from 362 patients treated between July 2007 to April 2019. We consecutively included all patients with clinical depression (leading to exclusion of 42 cases with an initial HDRS-17 total score < 8; defined as no depression^35^), with at least one completed HDRS-17 assessment from start of treatment until discharge (leading to exclusion of 31 cases) and treatment completion within a period of six weeks to 12 months (leading to exclusion of 50 cases). Patients with no HDRS-17 assessment did not differ from patients with assessment that were included in our analysis regarding age (t(359) = − 0.829, *p* = 0.408) and sex (Chi2(1) = 0.179, *p* = 0.673). This resulted in the final sample of 239 patients.

### Treatment

The day clinic comprised a multimodal treatment program for depression delivered by a multi-professional team consisting of consultant psychiatrists and resident psychiatrists, clinical psychologists, specialist nurses and occupational therapists. Treatment involved tailored individual and group psychotherapy of two sessions per week each. Individual psychotherapy included evidence-based psychodynamic and cognitive-behavioural psychotherapy provided by clinically experienced psychiatrists/residents in psychiatry and psychologists. Additional and supportive therapies, such as work-/occupational- and music therapy complement the treatment program. In line with current treatment guidelines, combination treatment, comprising a psychotherapeutic and psychopharmacological approach is common. The “dose” of all interventions is about 23–24 h of interventions per week (see figure S2 in the Supplementary material). Patients attend the day clinic five days per week. Treatment duration or discharge is decided by the responsible senior psychiatrist and the individual therapist together with the patient. Treatment duration is thus determined individually and can vary between patients.

### Measures

As part of the regular clinical care, patients report demographic details, including employment and civil status as well as level of education. Patients were screened by clinically experienced physicians and psychologists for depression and anxiety symptoms as part of the routine clinical documentation at admission, after six weeks of treatment and at discharge. Depression and anxiety symptoms were assessed using the clinician-administered Hamilton Depression Rating Scale (HDRS-17^36^) and the Hamilton Anxiety Rating Scale (HARS^37^). The HDRS-17 and HARS have extensively been used in research on depression^38^ and anxiety^39^. The reliability of the total depression symptoms (Cronbach’s α = 0.87) and the total anxiety symptoms (Cronbach’s α = 0.84) derived in our investigation were comparable to other studies^38,39^.

### Procedure

Assessments were conducted in a day clinic of an urban university teaching hospital offering treatment for patients diagnosed with depression. Treatment of depressive symptoms is the core focus, but patients’ diagnoses are not limited to major depressive disorders and comorbidity was frequent (see Table 1). Patients were screened for depression and anxiety symptoms as part of the routine clinical documentation at three time points, i.e., at admission, after six weeks of treatment and at discharge. All patients attended a clinical interview as part of the admission process. This included provision of ICD-10/DSM-IV diagnoses by trained raters supervised by a senior psychiatrist. Data were collected as part of the routine clinical care procedure and completely anonymized. In accord with local cantonal ethics guidelines and the Swiss Human Research act (HRA ^40^), no specific written informed consent was thus obtained.

**Table 1:** Sample and class characteristics.

	Responding from severe depression (n = 18)	Non-Responders (n = 47)	Responding from moderate severity (n = 174)	Total	X^2^ / F	*p* value
	M. (S.D.)	M. (S.D.)	M. (S.D.)			
Sex (% female)	10 (55.6%)	25 (53.2%)	100 (57.5%)	135 (56.5%)	.283	.868
Main diagnoses^a^						
MDD, single ep. (F32)	6	16	55	77	.111	.946
MDD, recurrent ep. (F33)	10	25	71	106	3.291	.193
Bipolar disorder, currently depressed (F31)	0	0	19	19	7.711	.017^g^*
F10–F19	0	0	2	2	.753	1.000^g^
F20–F29	0	0	2	2	.753	1.000^g^
F40–F49	1	4	21	26	1.052	.576^g^
F60–F69	1	2	4	7	.971	.494^g^
Age	42.5 (10.34)	40.9 (12.01)	41 (11.73)		.134	.875
Length of treatment (days)^c^	207.6 (110.41)	143.6 (79.52)	171.9 (86.93)		3.47	.041*
HDRS-17—Admission	30 (2.97)	21.87 (4.9)	15.42 (4.46)		111.55	.000^def^**
HDRS-17—After 6 weeks	23.61 (5.08)	21.44 (5.11)	13.17 (4.89)		77.22	.000^ef^**
HDRS-17—Discharge	14.31 (3.74)	22.37 (4.09)	7.55 (4.37)		227.84	.000^def^**
HARS—Total Value—Admission	22.7 (7.29)	18.65 (6.98)	13.34 (5.83)		28.296	.000^def^**
HARS—Somatic Anxiety—Admission^c^	9.1 (3.78)	6.48 (4.23)	4 (3.25)		20.06	.000^def^**
HARS—Psychic Anxiety—Admission	13.6 (4.62)	12.17 (3.79)	9.35 (3.67)		18.05	.000^def^**
Pat. w. comorbid diagnoses	10	30	78	118	5.64	.06
Comorbid diagnoses^a^						
F10–F19	6	8	47	61	.081	.261^g^
F20–F29	0	0	1	1	.38	1.000^g^
F30–F39	3	7	26	36	.04	1.000^g^
F40–F48	9	22	38	69	15.47	.000**
F50–F59	2	1	7	10	2.66	.264^g^
F60–F69	1	9	23	33	2.2	.333^g^
F70–F79	0	0	1	1	.38	1.000
F80–F89	0	0	0	0	–	–
F90–F98	0	1	8	9	1.39	.613^g^
Medication at admission^b^	15	41	150	206	.167	.920
Non-psychotropic drugs	7	20	49	76	4.36	.113
Antidepressants	15	36	137	188	.226	.929^g^
Anxiolytics	1	7	21	29	1.14	.555^g^
Detoxication/withdrawal	0	0	3	3	1,12	.689^g^
Hypnotics	3	4	5	12	8.07	.016^g^*
Neuroleptics	6	15	47	68	.904	.637
Mood stabilizers	5	9	23	37	3.37	.199^g^
Stimulants	1	1	5	7	.529	1.000^g^
Medication at discharge^b^	15	34	152	201	6.25	.044*
Non-psychotropic drugs	7	12	47	66	.42	.812
Antidepressants	15	26	110	151	1.27	.621^g^
Anxiolytics	1	2	11	14	.22	1.000^g^
Detoxication/withdrawal	0	0	3	3	1.09	.740^g^
Hypnotics	1	3	8	12	.79	.699^g^
Neuroleptics	4	12	33	49	3.74	.154
Mood stabilizers	5	10	32	47	1.4	.519^g^
Stimulants	2	1	6	9	1.52	.512^g^

MDD = Major depressive disorder; a Patients can have more than one comorbid diagnosis. b Patients can take more than one drug. c No homogeneity of variances—Welch ANOVA. d/e/f Significance tests (*p* = .05; Tukey or Games-Howell) between Resp./Non-Resp., Resp./Rem., Non-Resp./Rem., respectively. g Monte-Carlo estimation.

*p < .05. **p < .01.

F10–F19: Mental and behavioural disorders due to psychoactive substance use, F20–F29: Schizophrenia, schizotypal and delusional disorders,

F30–F39: Mood [affective] disorders, F40–F48: Neurotic, stress-related and somatoform disorders, F50–F59: Behavioural syndromes associated with physiological disturbances and physical factors, F60–F69: Disorders of adult personality and behaviour, F70–F79: Mental retardation, F80–F89: Disorders of psychological development, F90-F98: Behavioural and emotional disorders with onset usually occurring in childhood and adolescence.

The primary outcome of the predictive model was the grouping into non-responding patients vs. responding patients identified by LGMM. For model development of the LGMM, the outcome measure was the HDRS-17 scale, collected at admission, after six weeks of treatment and at discharge. Candidate predictor variables for the machine learning model were depression (HDRS-17) and anxiety (HARS; as mood and anxiety disorders are frequently comorbid and share symptoms^41^ and anxiety has been shown to distinguish within depression severity^42^) items at admission, as well as basic demographic variables, i.e., sex, age, civil status, level of education (none, elementary school, completed apprenticeship, secondary school, high school, university degree, other) employment before admission (fully employed, part-time employed, currently unemployed, unemployed).

### Statistical analysis

#### Latent growth mixture modelling of treatment response trajectories

To model heterogeneity in depression symptoms over the three time points, we employed LGMM using Mplus version 7 ^43^ to detect discrete growth trajectories (classes) and to test predictors of membership in these classes. Missing HDRS-17 total values were imputed using missForest_1.4 package in R^44^. This is an iterative imputation method based on random forests and can handle mixed data type containing both categorical and numerical variables^44^. It handles high-dimensional data containing complex non-linear relations as well as unequal variable scales and provides built-in out-of-bag estimates of the imputation error rate, which has been shown to be accurate for missing values ratio of up to 30%^44^ (please also refer to Figure S1 in the Supplementary material for a comparison of LGMM with imputed and non-imputed data). Missing values were ≤ 23% for the HDRS-17 total values during and at the end of treatment (all patients had admission HDRS-17 scores). LGMM handles errors as independent ^45^. LGMM identified heterogeneous trajectories based on depression symptoms at admission, after six weeks and at discharge. Individuals were assigned to trajectories based on their most likely class membership. For identifying the best-fitting model we followed recommendations from the literature^46^. We examined the Bayesian (BIC), sample size-adjusted Bayesian (SSBIC), and Akaike (AIC) information criterion indices, entropy values, the Lo-Mendell-Rubin likelihood ratio test (LMRT), and the bootstrap likelihood ratio test (BLRT) (see Table S1 in the Supplementary materials). Our aim was to find the best-fitting model with lower values for the criterion indices, higher entropy values, and significant p-values for both the LMRT and the BLRT. Our selection of the final model was determined by these indices, overall model fit, but also interpretability^47^.

#### Predictive modelling

In the first model, we predicted trajectories of depressive symptom course as outcome of a multinomial classifier (eXtremeGradient Boosting: XGBoost)^48^. In a second model, we built a binary classification model (XGBoost) to predict the two groups: "responding" (Responding from moderate severity) vs. "non-responding" trajectory (as the Responding from severe depression class was small and may be clinically different in terms of severity, often referred to as "very severe depression"^35,49^). XGBoost applies gradient descent optimization to minimize training error and is a tree-based ensemble method based on decision trees. It is a well-established and widely used machine learning approach due its great performance and high computational efficiency^48,50,51^. For data pre-processing, all numerical variables were normalized to range [0;1] and variables with near-zero-variance were removed using the built-in pre-process function from the caret R package^52^. Missing values were imputed using the missForest_1.4 package in R^44^. Missing values for the included variables in our sample were low (2%). To prevent “leakage” of information about the variable distribution in training and test set, we performed the pre-processing separately for the training and test set using caret. To maximize the likelihood of unbiased results, rigorous guards against over-fitting were implemented. First, the total data were randomly split into a 70% partition as training set and a 30% hold-out set to evaluate the predictive power of the final model in completely unseen new cases. To balance the dependent variable across data partitions, stratified random sampling was applied. During model training, 10 times repeated fivefold cross-validation was applied. For multi-class AUC we used the ‘pROC’ R package^53–55^. All analyses have been performed in R 3.5.3 using RStudio 1.2.1335.

#### Predictor importance ranking

Variables included in the final models were ranked with respect to their predictive power for the "responding" vs. the "non-responding" symptom trajectory memberships across the three assessments. We report methods for Explainable Machine Learning using SHAP (SHapley Additive exPlanation) values to examine and critically appraise on which features the model mainly relies to arrive at individual prediction outcomes. SHAP values were used to rank variables with respect to their ability to predict the "responding" vs. the "non-responding" trajectory^56^. This is an additive feature attribution method using kernel functions that enables consistent and locally faithful explanation of feature importance^56–58^.

### Ethics statement

Our study was conducted in accordance with the World Medical Association Declaration of Helsinki. Ethical approval was not required or obtained, since data were collected as part of the routine clinical care procedure and completely anonymized and did thus not fall under the Human Research Act (Humanforschungsgesetz).

## Results

Patients reported severe symptoms of depression, including significant dysfunction and impairment in their everyday life, i.e.: 33.9% received disability annuity, 57.8% were unable to work before treatment, 63.6% were considerably or severely ill according to ratings on a standardised seven items Likert scale of the Clinical Global Impression Scale ^59^ used in Swiss psychiatric hospitals as routine procedure, and more than 30% suffered from suicidal ideation. Overall, treatment was effective, but ineffective for a subgroup of patients (see below). Demographic and clinical sample characteristics are shown in Table 1.

### Identification of treatment response trajectories

The information indices and likelihood tests showed improved fit as the number of classes increased from one to four; however, this was not the case for BLRT, a more robust indicator^60^, which was not significant in the model with four classes. Also, the addition of a fourth class resulted in one very small class (two patients)—making the model less parsimonious and less interpretable. Consequently, the best fitting model was a three-class solution with a varying interval of the assessment at discharge (AIC = 4484.38, BIC = 4533.05, SSBI = 4488.67, VLMRT = 0.0063, BLRT = 0.0000) with a good entropy of 0.81 (see Supplementary Table S1). The most common symptom trajectory was a "responding" (responding from moderate severity) class, starting from a moderately depressed level to a level below clinical depression^61^ (n = 174; 72.8%), followed by a “non-responding” class, starting from a moderately to severely depressed level^35,49^ (mean total value of HDRS-17 at admission = 21.9; n = 47; 19.7%) and no symptom improvement over time (mean total value of HDRS-17 at discharge = 22.3). A third class was less common, “Responding from severe depression", starting from a severely depressed level (mean total value of HDRS-17 at admission = 30) to improvement (mean total value of HDRS-17 at discharge = 14.3) although on average not reaching complete remission (n = 18; 7.5%).

**Figure 1:**
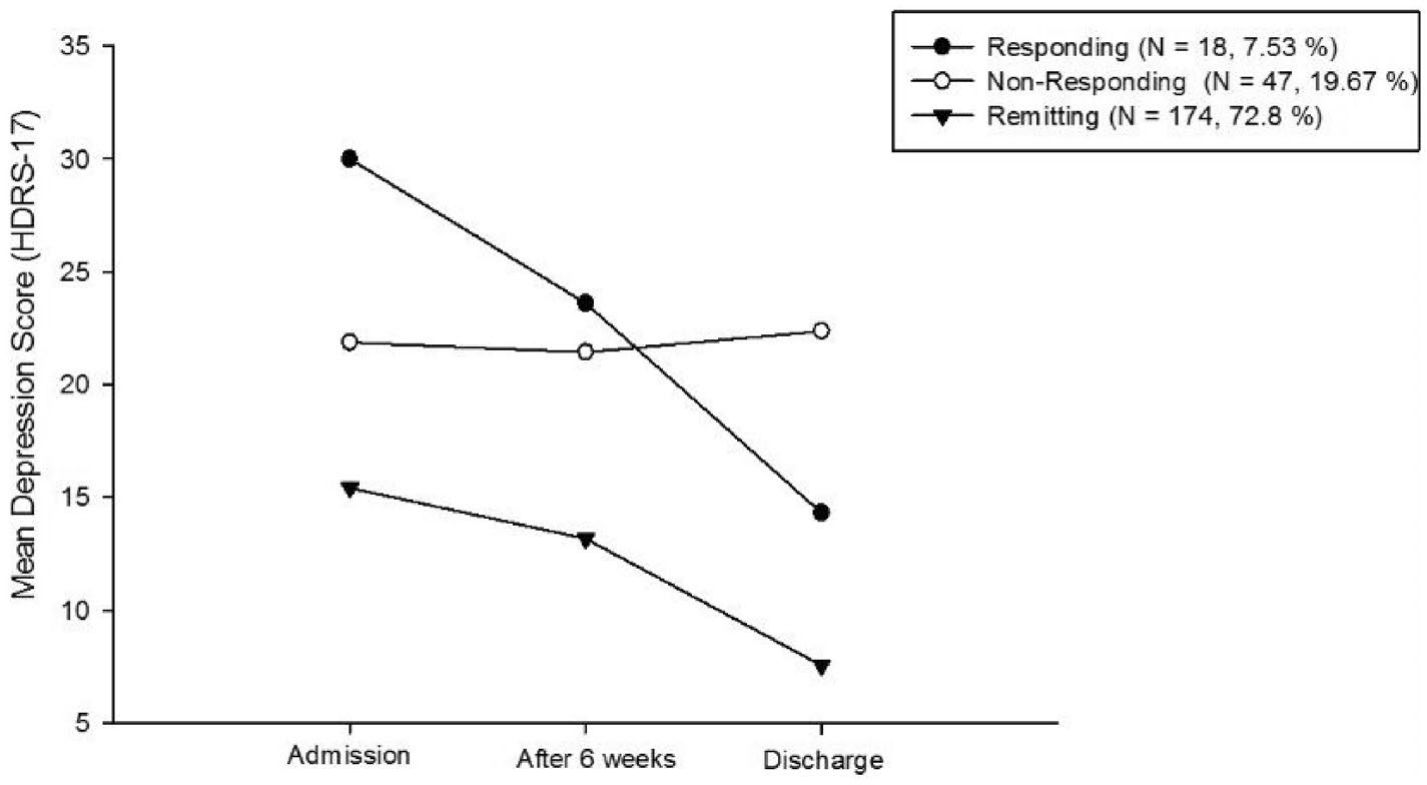
Mean depression score as a function of time point of assessment and class (*N* = 239). Depression was rated using the Hamilton Depression Rating Scale^1^; higher numbers indicate greater depression levels.

The unconditional LGMM is shown in Fig. 1. The "non-responding" vs. the "responding" class memberships were used as the outcome for XGBoost.

### Predicting response trajectories from baseline clinical and demographic indices

**Figure 2:**
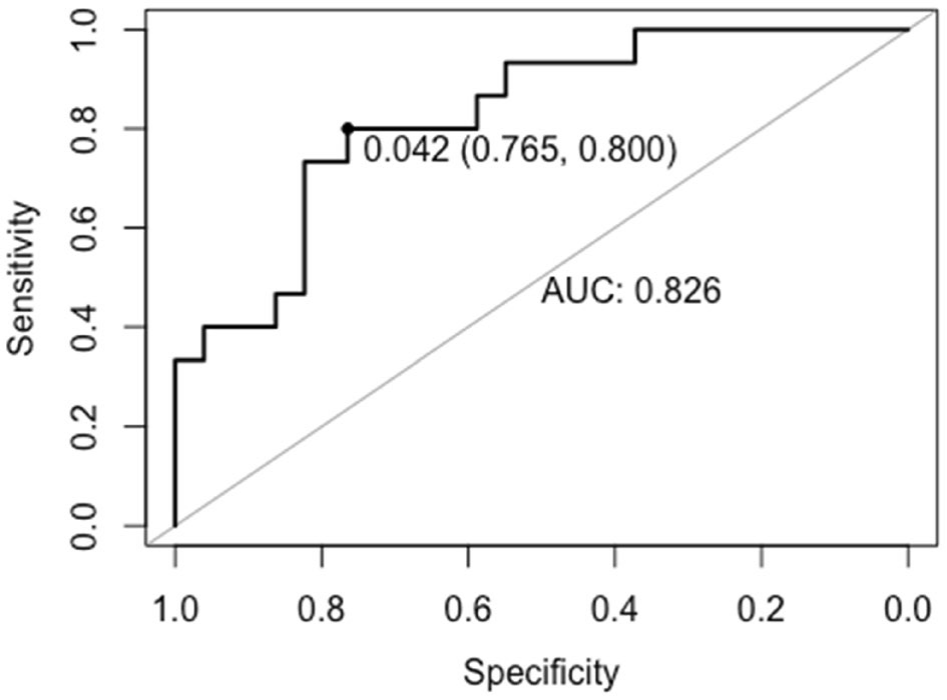
Receiver Operating Characteristic (ROC) curve for the binary classification evaluating the predictive power in the hold-out set. Optimal ROC threshold with the highest sum of sensitivity + specificity is plotted with specificity followed by sensitivity in brackets^2^.

The XGBoost algorithm for predicting all three symptom trajectories yielded a multi-class AUC = 0.80 in the hold-out set. The predictive power for the binary classification (XGBoost) for discriminating the "responding" and "non-responding" trajectory was AUC = 0.83 (sensitivity = 0.80, specificity = 0.77) (see Fig. 2).

### Ranking predictor variables for predictive value

**Figure 3:**
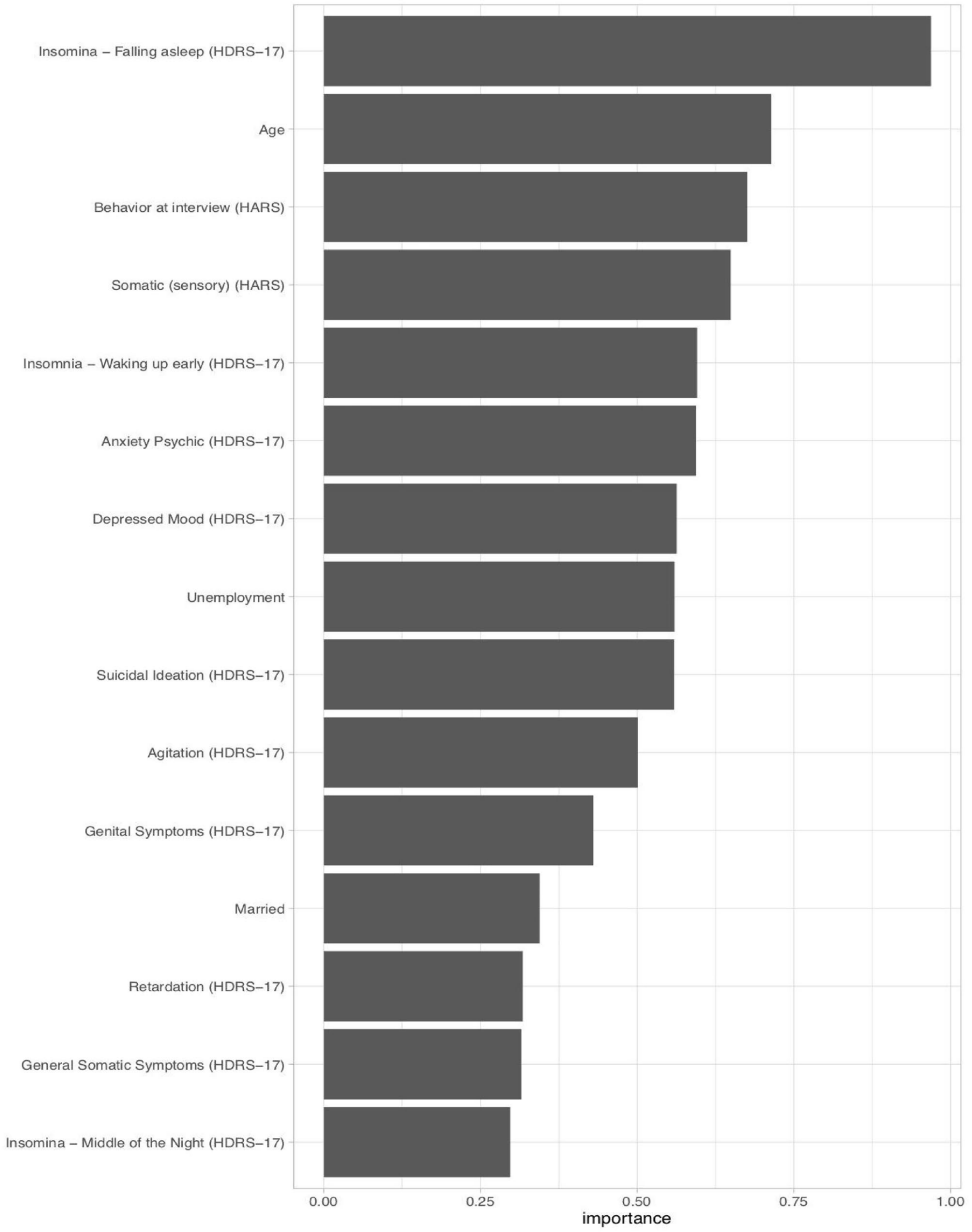
Variable importance for the hold-out set using SHAP (SHapley Additive exPlanations)^3^. Presented are the 15 most influential features in predicting “responding” vs. “the non-responding” symptom trajectory memberships.

**Figure 4:**
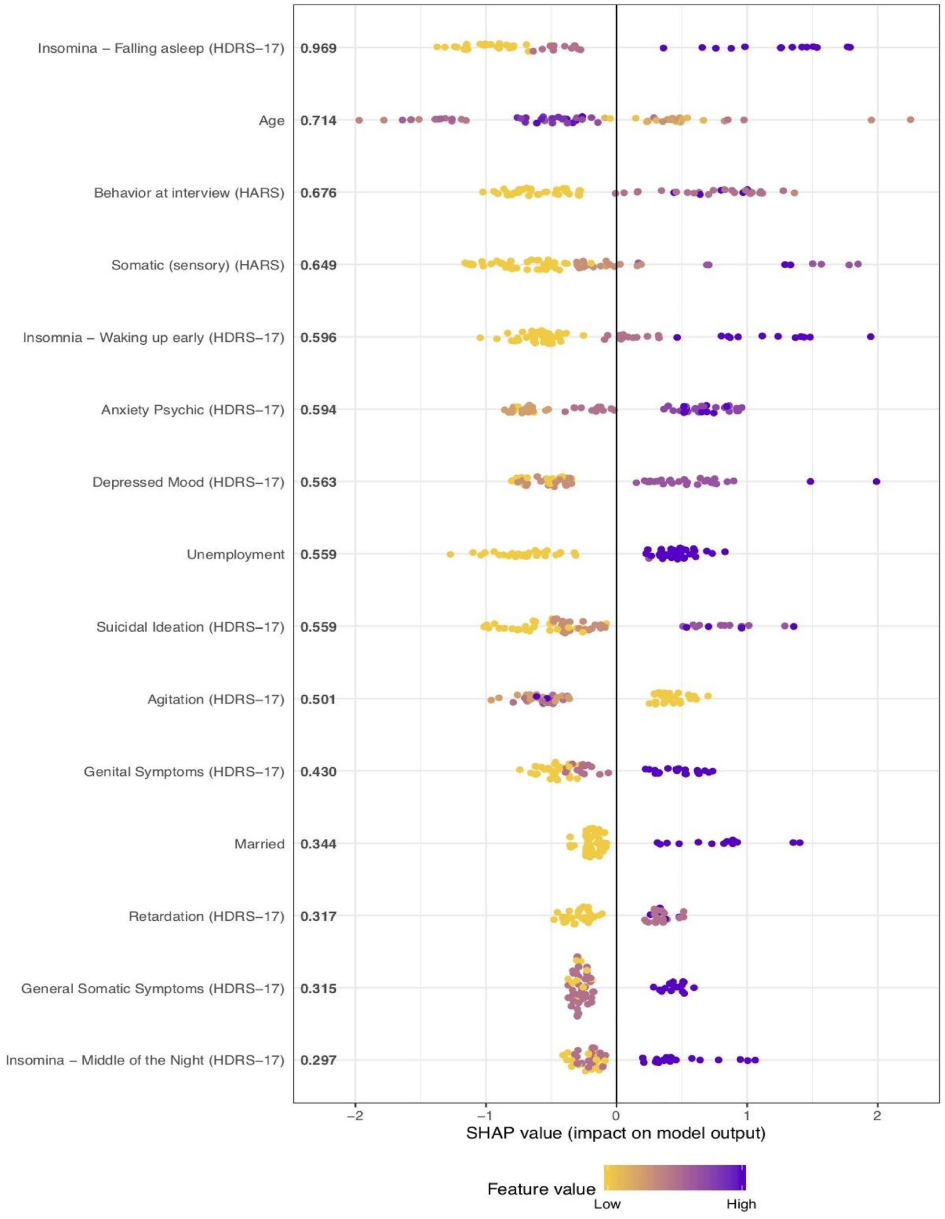
SHAP summary dot plot, displaying, which features influence the model predictions of the “non-responding” trajectory the most. The higher the SHAP value of a feature, the higher the log odds of a “non-responding” depression trajectory.

**Figure 5:**
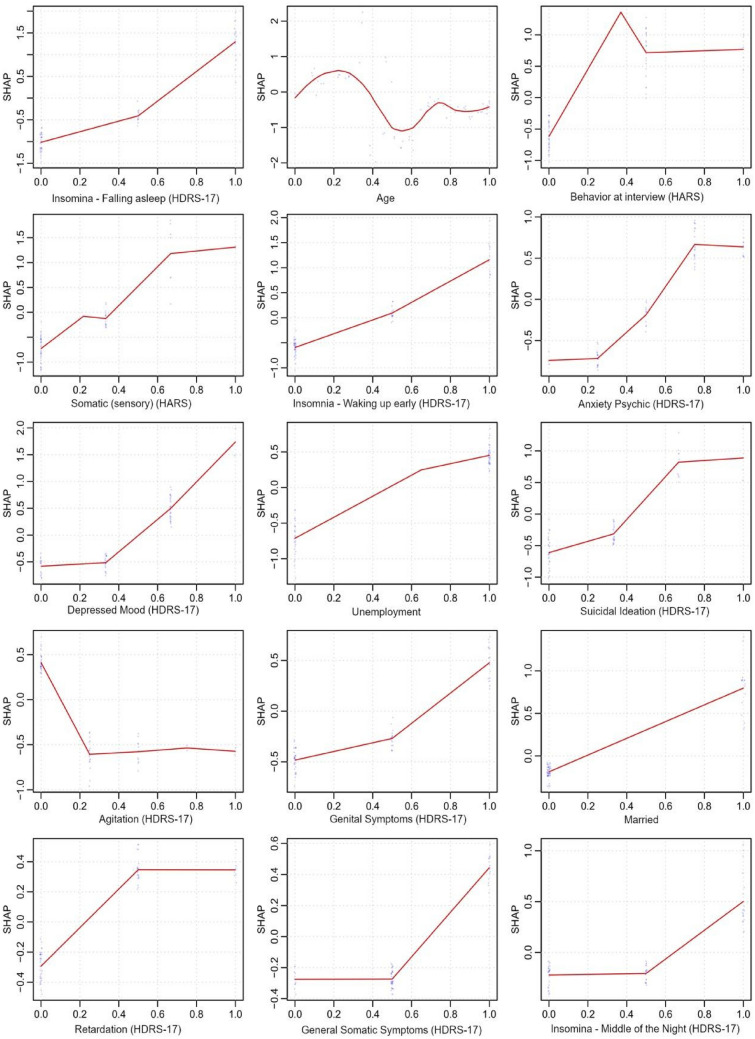
SHAP values for the hold-out set. This figure displays the decision rule for each feature for predicting “responding” vs. the “non-responding” symptom trajectory memberships.

Figure 3 displays the predictor variable importance rankings using SHAP values^56^. The strongest features predicting the "responding" vs. the "non-responding" symptom trajectory memberships included mainly HDRS-17 items and some demographic characteristics: *Insomnia—Falling asleep*, younger age, *Behaviour at interview* (HARS), *Somatic (sensory) anxiety* (HARS), *Insomnia—Waking up early*, *Anxiety Psychic*, *Depressed Mood*, employment status (unemployed), *suicidal ideation*, lower levels of *Agitation*, *Genital symptoms*, *being married*, *Retardation*, *General Somatic symptoms*, and *Insomnia—Middle of the Night*. For additional information on the variable importance rankings using SHAP values see Figs. 4 and 5.

## Discussion

We identified heterogeneity in longitudinal trajectories of depression symptoms over the course of multimodal treatment using LGMM in a day clinic. Specifically, we identified treatment response trajectories resulting in a three-class solution comprising a “non-responding” subgroup (with no symptom improvement over the course of treatment), a “Responding from severe depression" subgroup (responding from severe depression to a level of moderate depression) and a "responding" subgroup (responding from moderate depression to a level below clinical depression)^61^. All groups were severely impaired when they started treatment at the day clinic. Both "Responding from severe depression" and "responding" (Responding from moderate severity) subgroups showed overall decline in their depression symptom severity of 50% or more at discharge compared to admission. The "non-responding" class showed no change in depression symptoms. Length of stay within the non-responder group was shorter on average (Welch's F(2,39.946) = 3.473, *p* = 0.041), since some patients may have been transferred to an inpatient ward.

Next, we applied ML to predict these trajectories. Since we were most interested in differentiating non-response from response, we predicted group membership for the "non-responding" compared to the "responding" group using XGBoost^48^. SHAP Values^56^ identified insomnia (problems falling asleep being the most predictive item), younger age, anxiety symptoms, depressed mood, being unemployed, suicidal ideation and several somatic indicators of depressive disorders as most important predictors for non-response. The overall prediction model yielded high predictive power. These outcomes compare favourably to other studies using similar models in treatment outcome prediction^28,62–64^.

The model identified sleep disturbances as one of the top predictors of poorer outcome, i.e., non-response compared to remitting depression symptoms. Patients reporting sleep problems initially were more likely to be in the non-responding group. In line with our findings, Troxel et al.^65^ showed that problems falling asleep significantly increased the risk of non-remission following pharmacologic and/or psychotherapeutic treatment for depression and identified this symptom as one of the strongest predictors in their study. Basic neuroscience studies consistently link sleep to memory, learning, and, in general, to the mechanisms of neural plasticity^66^. An increasing body of evidence shows that sleep plays a pivotal role in the orchestration of neuroplasticity^67^. Such processes are paramount for processing and benefiting from psychotherapeutic treatment, which was a fundamental pillar of treatment in the day clinic treatment investigated in this study. Sleep problems may thus have resulted in decreased plasticity and capacity to learn during psychotherapy and consequently increased the probability of non-response to treatment. Together, these results link sleep to the recovery processes and suggest a target for depression treatment. Improving sleep, for instance with cognitive-behavioural treatment approaches, may lead to increased plasticity, capacity to learn and process (emotional) memories and thus benefit from treatment^66,68^.

Suicidal ideation at admission was another indicator of non-response to depression treatment. The current data were collected as part of routine clinical care, hence including a significant proportion of patients with suicidal symptoms such as thoughts and ideations, a group often formally excluded from randomised controlled treatment trials^69^. Our model results thus include relevant information regarding this group, highlighting a subgroup of patients in need of specific attention and potentially augmented treatment regimens^70^. Whilst there are only few evidence-based treatment programs for suicidal psychiatric patients, potential options exist, including for instance, cognitive therapy interventions designed to prevent repeat suicide attempts in adults who recently attempted suicide^71^ or dialectical behaviour therapy, which was also shown to be effective in reducing suicide attempts^72^.

In line with recent studies underlining the impact of social and economic risks associated with neighbourhood safety, educational attainment, housing stability on mental health outcomes^73^, demographics were also ranked as key predictors in our dataset. Interestingly, and in contrast to a previous study that predicted course trajectories of depressed outpatients^74^, younger age was associated with heightened probability of being a non-responder. Unfortunately, we do not have detailed information on the age of first depressive symptoms, but previous studies have associated the early-onset subtype of depression with worse outcomes overall^74–76^. Unemployment, the second most predictive demographic index, may be a risk for mental health or the result of it^77–79^ and hamper effects of psychotherapy^80,81^. Marriage has been shown to positively affect well-being^82^, but may also be associated with negative consequences and individual, interpersonal, and structural features that may impede recovery^83^. Sex was not amongst the top 15 predictors and this is in line with previous studies^20,84^, but also see^74,85^.

There are several limitations to the study. First, exact information on number and severity of previous depressive episodes and duration of the current depressive episode was not available and their predictive value thus remains to be tested in such predictive models^20,23,86^. Second, we examined a rather small spectrum of predictors (e.g., no biological markers, e.g.^87^). However, if replicated across other samples and treatment trajectories, the items assessed as part of this study and those selected by the models are easily integrated into routine clinical practice. Third, in LGMM, class membership assignments differ naturally in accuracy for individual patients and were accurate for some patients and less precise for others. Fourth, data were collected as a routine clinical quality assessment and provide naturalistic data on heterogeneity in treatment responses and their prediction for those patients treated at the day clinic for 6 weeks to 12 months. We replicated the prediction in the holdout sample within our dataset; a reproduction nevertheless is pending for other samples. Fifth, a structured Axis II-diagnostics is missing. Sixth, within-treatment characteristics (for which there are no data available, e.g., change of medication or adherence to psychotherapy) may differ between patients and between trajectory classes and should be included in future studies. Seventh, a larger sample size would enable more comprehensive computations. Finally, response trajectories relied on measurements at three time points. More measurements would allow for more detailed assessments of trajectories (e.g.,^88^) and this may also require larger sample sizes for their identification. Future studies could expand on our findings and test whether self-report questionnaires of the same symptoms derive at the same results providing easier data collection. These data can also be used within a prediction model to help dynamically enhance psychotherapy outcomes^89^. These limitations are met by several key strengths. The naturalistic study design made use of a consecutive sample, warranting high external validity. Since treatment was comparable for all patients, internal validity was acceptable. Additionally, the longitudinal design reveals information about treatment outcome. Also, we only used clinician administered instruments, conducted by psychiatrists/residents in psychiatry or psychotherapists.

Taken together, we identified heterogeneity in multimodal treatment outcome in depressed psychiatric patients. A predictive algorithm based on basic clinical and demographic data obtained in routine clinical practice identified treatment non-responders from those who responded during treatment with high predictive accuracy. These results have clinical relevance as the items selected by our algorithm could be easily obtained in clinical practice. In this heterogeneous sample of patients presenting with depression, our model was able to predict response versus non-response to multimodal treatment. Given replication of our results in other clinical settings, and possibly other groups and health care systems, such early predictors of treatment response could help pave the way towards more effective personalized therapeutic approaches and optimize treatment outcomes.

## Supplementary Information

Supplementary Information.
