# Supplementary material for: Predicting non-response to multimodal day clinic treatment in severely impaired depressed patients: a machine learning approach
Source: Sci Rep. 2022 Mar 31;12:5455. doi: 10.1038/s41598-022-09226-5 (PMC8971434; doi:10.1038/s41598-022-09226-5)
Supplement: Supplementary file 1 — Supplementary Information. [file 41598_2022_9226_MOESM1_ESM.docx]

Supplementary materials for

**Predicting non-response to multimodal day clinic treatment in severely impaired depressed patients: A machine learning approach**

Johannes Simon Vetter^1*^, Katharina Schultebraucks^2,3*^, Isaac Galatzer-Levy^4^, Heinz Boeker^1^, Annette Brühl^1^, Erich Seifritz^1^, Birgit Kleim^1^

^1^Psychiatric University Hospital, University of Zurich, Zurich, Switzerland

^2^Vagelos School of Physicians and Surgeons, Department of Emergency Medicine, Columbia University Medical Center, New York, NY, USA

^3^Department of Psychiatry, Columbia University

^4^Department of Psychiatry, NYU Grossman School of Medicine, New York, New York

* These authors contributed equally to this paper

The tables and figures presented in this supplementary materials file are fit indices for the Latent Growth Mixture Models of Depression Symptom Severity (N = 239). The analyses were conducted using Mplus ^1^, following the recommendations of van de Schoot et al. (2017) ^2^.

**Table S1.** Fit Indices for the Latent Growth Mixture Models^a^ of Depression Symptom Severity (N = 239)

|  | Model | | | |
| --- | --- | --- | --- | --- |
| Fit index | Two classes | Three classes | Four Classes | Five classes |
| AIC | 4501.527 | 4484.376 | 4480.659 | 4477.978 |
| BIC | 4539.768 | 4533.046 | 4539.759 | 4547.507 |
| SSBIC | 4504.902 | 4488.670 | 4485.874 | 4484.113 |
| BLRT | <0.001 | <0.001 | 0.1176 | 0.2273 |
| VLMRT | 0.0260 | 0.0063 | 0.0365 | 0.3589 |
| Entropy | 0.767 | 0.812 | 0.852 | 0.852 |
| Sample Size Per Class^b^ | 173/66 | 18/47/174 | 169/19/49/2 | 2/118/62/40/17 |

^a^ Variance of HDRS-17 total value at discharge fixed to zero due to negative residual variance (which was small and insignificant)

^b^ Based on Most Likely Class Membership

AIC = Akaike information criterion; BIC = Bayesian information criterion; SSBIC = sample-size-adjusted Bayesian information criterion; BLRT = bootstrap likelihood-ratio test; VLMRT = Vuong-Lo-Mendell-Rubin likelihood ratio test

**Supplementary section for Table 1.**

Post-hoc analyses revealed that significantly more patients with anxiety, dissociative, stress-related, somatoform and other nonpsychotic mental disorders (ICD-10 F4; ^3^ as the main diagnosis were in the remitting subgroup of patients (s. Table 1). While also suffering from a depressive syndrome, anxiety symptoms may have been more central in some of our patients afflicted with comorbid depressive disorders and can be treated with faster response. As several indicators of anxiety predicted worse outcomes in line with other studies ^4,5^, on the other hand, the subgroup of depressed patients with high levels of anxiety ^6^ may need special attention. Also, the responding and non-responding classes had significantly higher levels of somatic and psychic anxiety (s. Table 1). Mood and anxiety disorders are indeed frequently comorbid and share symptoms ^7^ and anxiety has been shown to distinguish within depression severity ^8^. From an etiological perspective, anxiety symptoms often precede depression ^9,10^, and are associated with worse depression outcomes ^11,12^. Additionally, anxiety has been identified as a factor that may hamper efficacy of psychotherapy for depression ^13,14^ and facilitate chronification ^15^. In line with Paul *et al.* (2019), our findings stress the importance of targeting anxiety symptoms in the treatment of depression and may call for personalized psychotherapy approaches.

**Figure S1.** Sample means trajectory plots

A: Trajectories based on imputed data

B: Trajectories based on complete assessment data

Both LGMM models have an equal Entropy of 0.812, demonstrating a fairly good classification quality ^2^. Moreover, the trajectories are highly similar and differ only minimally in the percentage of how many participants are in each group.

**Figure S2. Weekly Schedule – Day Clinic**


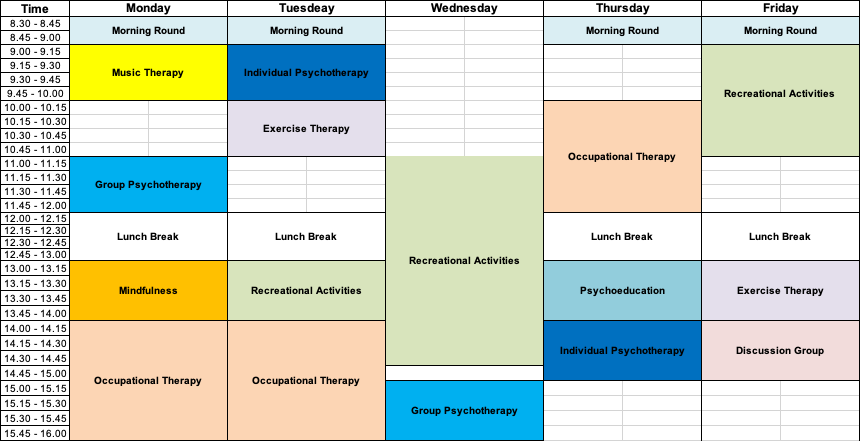


**References**

1. Muthén, L. K. & Muthén, B. O. *Mplus User’s Guide. Eighth Edition*. (Muthén & Muthén, 1998).

2. van de Schoot, R., Sijbrandij, M., Winter, S. D., Depaoli, S. & Vermunt, J. K. The GRoLTS-checklist: Guidelines for reporting on latent trajectory studies. *Struct. Equ. Model.* **24**, 451–467 (2017).

3. World Health Organization. *ICD-10: international statistical classification of diseases and related health problems*. (World Health Organization, 2004).

4. Huibers, M. J. H. *et al.* Predicting Optimal Outcomes in Cognitive Therapy or Interpersonal Psychotherapy for Depressed Individuals Using the Personalized Advantage Index Approach. *PLoS ONE* **10**, (2015).

5. Paul, R. *et al.* Treatment response classes in major depressive disorder identified by model-based clustering and validated by clinical prediction models. *Transl. Psychiatry* **9**, 1–15 (2019).

6. Baumeister, H. & Parker, G. Meta-review of depressive subtyping models. *J. Affect. Disord.* **139**, 126–140 (2012).

7. Kotov, R. *et al.* New Dimensions in the Quantitative Classification of Mental Illness. *Arch. Gen. Psychiatry* **68**, 1003–1011 (2011).

8. ten Have, M. *et al.* The identification of symptom-based subtypes of depression: A nationally representative cohort study. *J. Affect. Disord.* **190**, 395–406 (2016).

9. Ionescu, D. F., Niciu, M. J., Richards, E. M. & Zarate, C. A. Pharmacologic Treatment of Dimensional Anxious Depression: A Review. *Prim. Care Companion CNS Disord.* **16**, (2014).

10. Uher, R. *et al.* Melancholic, atypical and anxious depression subtypes and outcome of treatment with escitalopram and nortriptyline. *J. Affect. Disord.* **132**, 112–120 (2011).

11. Coryell, W. *et al.* Effects of anxiety on the long-term course of depressive disorders. *Br. J. Psychiatry* **200**, 210–215 (2012).

12. Goldberg, D. & Fawcett, J. The importance of anxiety in both major depression and bipolar disorder. *Depress. Anxiety* **29**, 471–478 (2012).

13. Fava, M. *et al.* Background and rationale for the Sequenced Treatment Alternatives to Relieve Depression (STAR∗D) study. *Psychiatr. Clin. North Am.* **26**, 457–494 (2003).

14. Reich, J. *et al.* Comorbidity of panic and major depressive disorder. *J. Psychiatr. Res.* **27 Suppl 1**, 23–33 (1993).

15. van Loo, H. M. *et al.* Major depressive disorder subtypes to predict long-term course. *Depress. Anxiety* **31**, 765–777 (2014).
